# Pediatric paraparesis: Radiological challenges in multidrug-resistant tuberculous spondylitis based on experience in Indonesia

**DOI:** 10.1016/j.radcr.2024.04.001

**Published:** 2024-05-18

**Authors:** Damayanti Sekarsari, Quinta Febryani Handoyono, Mohamad Yanuar Amal, Maria Angela Putri Maharani, Andra Hendriarto

**Affiliations:** a Division of Pediatric Radiology, Department of Radiology, Faculty of Medicine, University of Indonesia, Dr. Cipto Mangunkusumo General Hospital, Jl. Salemba Raya No. 4, Senen, Central Jakarta, DKI Jakarta; b Department of Radiology, Faculty of Medicine, University of Indonesia, Dr. Cipto Mangunkusumo General Hospital, Jl. Salemba Raya No. 4, Senen, Central Jakarta, DKI Jakarta; c Department of Anatomic Pathology, Faculty of Medicine, University of Indonesia, Dr. Cipto Mangunkusumo General Hospital, Jl. Salemba Raya No. 4, Senen, Central Jakarta, DKI Jakarta; d Department of Orthopedics and Traumatology, Faculty of Medicine, University of Indonesia, Dr. Cipto Mangunkusumo General Hospital, Jl. Salemba Raya No. 4, Senen, Central Jakarta, DKI Jakarta

**Keywords:** Tuberculous spondylitis, Tuberculosis, Pediatric imaging

## Abstract

Multidrug-resistant tuberculous spondylitis is a global health issue, especially in developing nations, and non-specific symptoms lead to delay in identification, treatment, and potential disability in children. Radiology plays a crucial role in diagnosing tuberculous spondylitis, which in turn might lead to effective treatment, prevention of disability and improved patient outcomes. Our case involved a 20-month-old malnourished child presented with paraparesis, revealing a history of contact with parental multidrug-resistant tuberculosis. Multimodality radiological examinations, including conventional radiography, CT, and MRI revealed extensive disease of the spine with disc involvement, large paravertebral abscess, and kyphotic deformity which produced neurological deficits, necessitating both anti-tuberculosis regimen and surgical intervention. Radiological examinations have a pivotal role in diagnosing, evaluating and guiding timely management of multidrug-resistant tuberculous spondylitis. Prompt diagnosis of the condition is crucial in order to prevent potentially severe complications, which contribute significantly to morbidity. Our case demonstrated the importance of radiology in diagnosing extensive spine involvement of the disease causing neurological deficits. Furthermore, radiology also helps in managing tuberculous spondylitis to prevent future disability in a child patient of a developing country. This case highlights the crucial significance of radiological imaging in the diagnosis and management of pediatric tuberculous spondylitis in impoverished nations. The patient's complex medical history highlights the socioeconomic factors contributing to tuberculosis burden. Early and comprehensive radiological assessment, together with collaboration between radiologists and clinicians, is vital for timely intervention and improved outcomes in pediatric tuberculous spondylitis cases to prevent the impact of this debilitating disease on children.

## Background

Tuberculosis, caused by the slow-growing aerobic bacteria Mycobacterium tuberculosis, is a prevalent infectious disease worldwide and remains a significant global health concern [1,2]. According to the World Health Organization, approximately 10.6 million individuals were infected with tuberculosis in 2022, with 1.3 million of them being pediatric patients [3,4]. Children are still vulnerable due to the presence of frequently non-specific symptoms, which can cause a delay in both diagnosis and treatment [2]. This disease, its disability, and mortality in the pediatric population can be treated and prevented, particularly in low-income nations [4,5]. However, in developing countries with a substantial burden like Indonesia, there is still a notable occurrence of childhood tuberculosis, representing approximately 10%-20% of all tuberculosis (TB) cases [2,6]. Indonesia experienced a significant increase in childhood tuberculosis cases, rising from 42.187 in 2021 to 100.726 in 2022, indicating a more than 200 percent increase [7]. In 2022, there were also 2.2 million new cases of tuberculosis worldwide caused by undernutrition, and 0.89 million cases caused by HIV infection [4]. Additionally, multidrug-resistant tuberculosis (MDR-TB) remains as a public health emergency, with children at higher risk and a treatment-seeking rate of only 2 in 5 patients in 2022 [4,8]. It is primarily caused by improper medication use, such as inaccurate prescriptions and premature therapy discontinuation, which at the end makes the management of extensive resistance difficult [4].

While tuberculosis primarily targets the lungs and mediastinal lymph nodes, it can affect any organ system; termed extrapulmonary tuberculosis [5]. Extrapulmonary tuberculosis, specifically musculoskeletal TB, is more prevalent in children compared to adults [2]. Tuberculous spondylitis is the predominant type of musculoskeletal tuberculosis in children, representing over 50% of the cases [5,9]. Pediatric tuberculous spondylitis typically manifests with a gradual onset, slow progression, and mild initial symptoms [2]. This poses a diagnostic challenge as it makes it difficult to identify the disease in its early stages, leading to a delay in therapy [2,9]. Untreated spinal tuberculosis can rapidly progress, leading to nerve compression, spine abnormalities, and disabling complications, underscoring the need for prompt diagnosis and intervention to prevent severe damage to the bone, joints, surrounding muscles, and soft tissues [1,5].

In addition to the patient's clinical history, physical examination, and laboratory examinations, a range of radiological examinations are used to confirm the diagnosis [5]. Radiology plays a crucial role in diagnosing tuberculous spondylitis, which in turn might lead to effective treatment, prevention of disability and improved patient outcomes [2]. Each imaging modalities has a distinct role in diagnosing and comprehensively evaluating tuberculous spondylitis, which helps determine the most appropriate treatment approach for each patient. In this report, we present a case of multidrug-resistant tuberculous spondylitis in a 20-month-old malnourished child. We discuss the impact of the disease on the child's ability to function and emphasize the significance of conducting a thorough radiological assessment and implementing appropriate treatment in the setting of a developing nation, which ultimately improved the patient's condition.

## Case report

A 20-month-old child was referred to our facility due to paresis in both legs since 1 month before admission. Previously, the patient had ambulatory capabilities without any prior history of trauma, seizure, or any other neurological deficits. Both upper extremities were functioning normally. Prior to the patient's paraparesis, she was at first encountering ambulatory challenges. She was walking with a pronounced sideways tilt of her torso and a dragging gait. The patient's lower extremities became weaker as the disease progressed. Eventually, she lost the ability to walk, which prompted her referral to our facility.

The guardian also noticed a mass on her midback, which was first noticed 1 month prior to admission, although information describing the growth of the mass was insufficient. Another observed sign was the absence of weight gain in the patient over the preceding 6 months. The patient denied any past occurrences of cough, fever, night sweats, or any other past medical history. The patient's older sibling was diagnosed with pulmonary tuberculosis and had just completed his 6-month anti-tuberculosis regimen. Furthermore, both of the patient's parents passed away as a result of MDR TB. Due to the patient's orphan status, obtaining comprehensive information about her birth, growth, and development history was not possible. At the first presentation at a different medical facility, the patient was diagnosed with probable spinal TB. Subsequently, she has been undergoing treatment with anti-tuberculosis therapy (ATT) for a duration of 1 month prior to her admission at our facility.

Upon physical examination, the patient was alert and afebrile. Nutritional status was evaluated based on the WHO growth chart, indicating malnutrition with a weight of 8 kg and height of 76 cm. The vital signs and examinations of the anterior thoracic region, as well as the lymphatic nodes, were within normal limits. The neurological examination showed a bilateral positive Babinski and Clonus reflex, as well as a muscle strength score of 1 in the lower extremities and 5 in the upper extremities, with increased physiological reflexes, especially in the lower extremities. The patient did not exhibit any paresthesias. A kyphotic deformity was observed in the posterior thoracic region, accompanied by a mass on the midback region. The mass had a diameter of approximately 10 cm and was warm and painful to touch. A laboratory investigation showed anemia with Hb of 8.1 g/dL, leukocytes of 6.350 u/L, hyponatremia of 128 mEq/L, hypochloremia of 95.3 mEq/L, increased C-reactive protein (CRP) of 18.3 mg/dL, and an increased erythrocyte sedimentation rate (ESR) of 71 mm. An HIV screening test was conducted and yielded a negative result. It was not possible to collect a sputum sample for acid-fast bacilli examination.

**Fig. 1:**
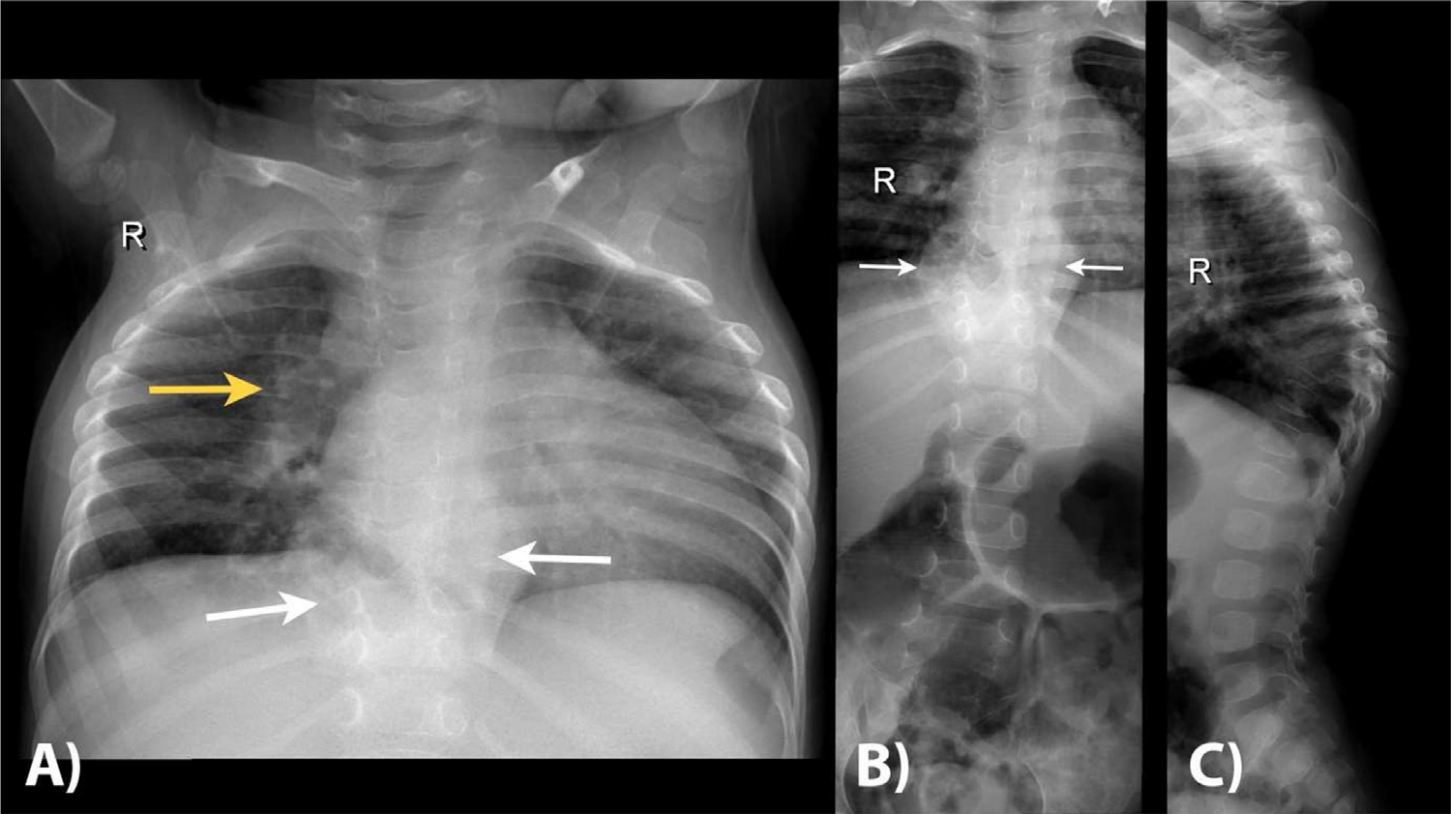
A conventional radiograph of the thorax showed right perihilar pulmonary consolidation (yellow arrow in A), Gibbus formation at the level of T8-10 and soft tissue mass appearance at the level of T5-11 (white arrows on A). The thoracolumbar radiograph confirmed the presence of vertebral bodies destruction and paravertebral soft tissue mass between the T5-11, suggesting the diagnosis of tuberculous spondylitis with paravertebral abscess (arrows in B and C).

The patient underwent a series of radiological examinations to assess her condition further. An initial conventional radiograph of the thorax showed right perihilar pulmonary consolidation, Gibbus formation at the level of T8-10, and a soft tissue mass appearance at the level of T5-11 (Fig. 1A). The thoracolumbar radiograph confirmed the presence of vertebral bodies destruction and paravertebral soft tissue mass between the T5-11, suggesting the diagnosis of tuberculous spondylitis with paravertebral abscess (Figs. 1B and C).

**Fig. 2:**
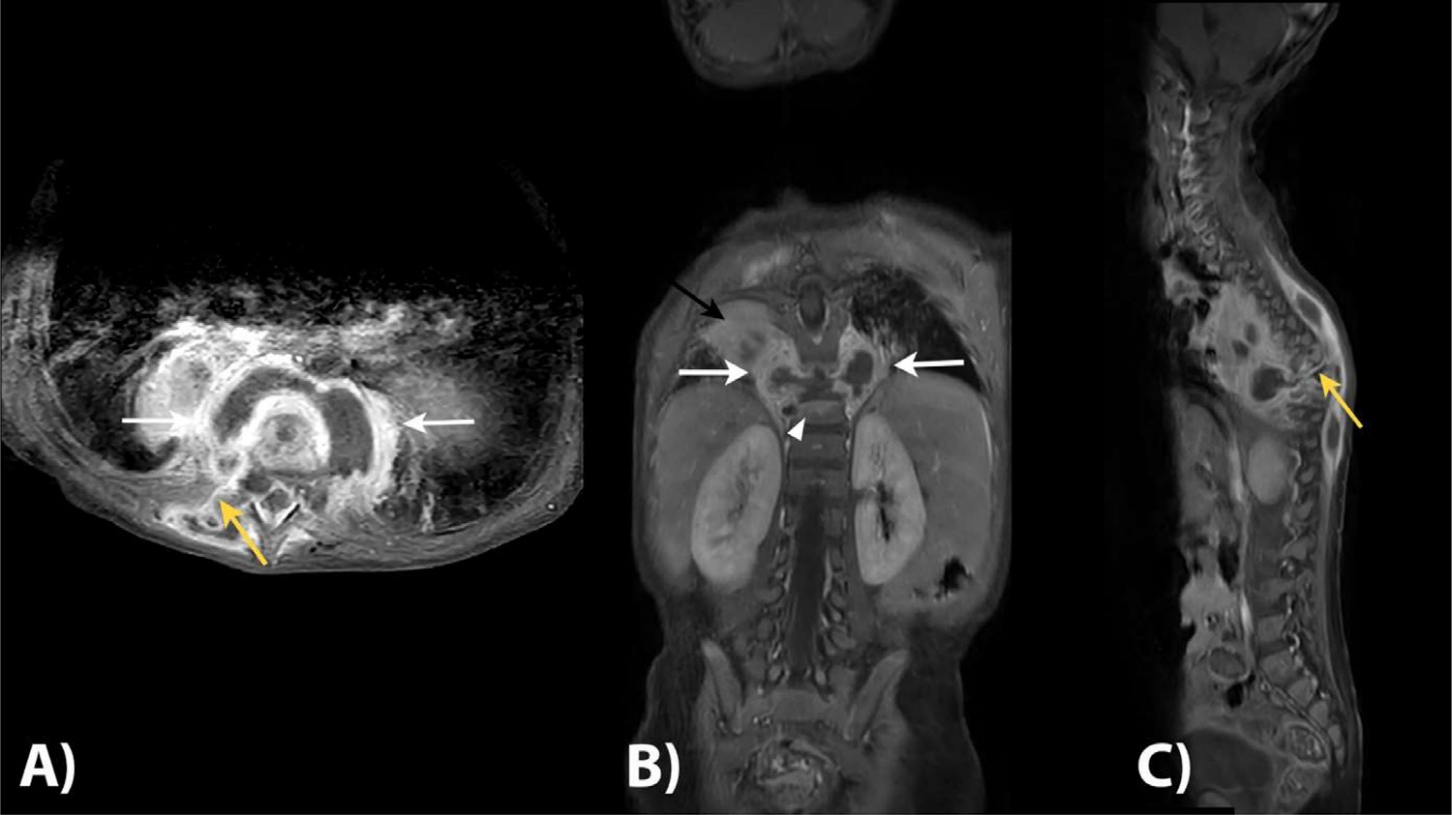
Contrast-enhanced spinal MRI with T1W and T1W FS sequences showed spondylodiscitis affecting the T8-10 vertebrae, which resulted in the destruction of the vertebral bodies and a kyphotic deformity at that level (white arrows in Figure 2). It also revealed the presence of a bilateral paravertebral abscess, which formed a fistula extending to the right erector spinae muscle and the subcutaneous soft tissue starting from the T3-11 level (marked by yellow arrow in A and C). Additionally, B and C showed that the anterior portion of the spinal canal at the T7-11 level was affected, and there was swelling of the bone marrow along the vertebral bodies of T8 and T10 (marked with arrowhead in B).

**Fig. 3:**
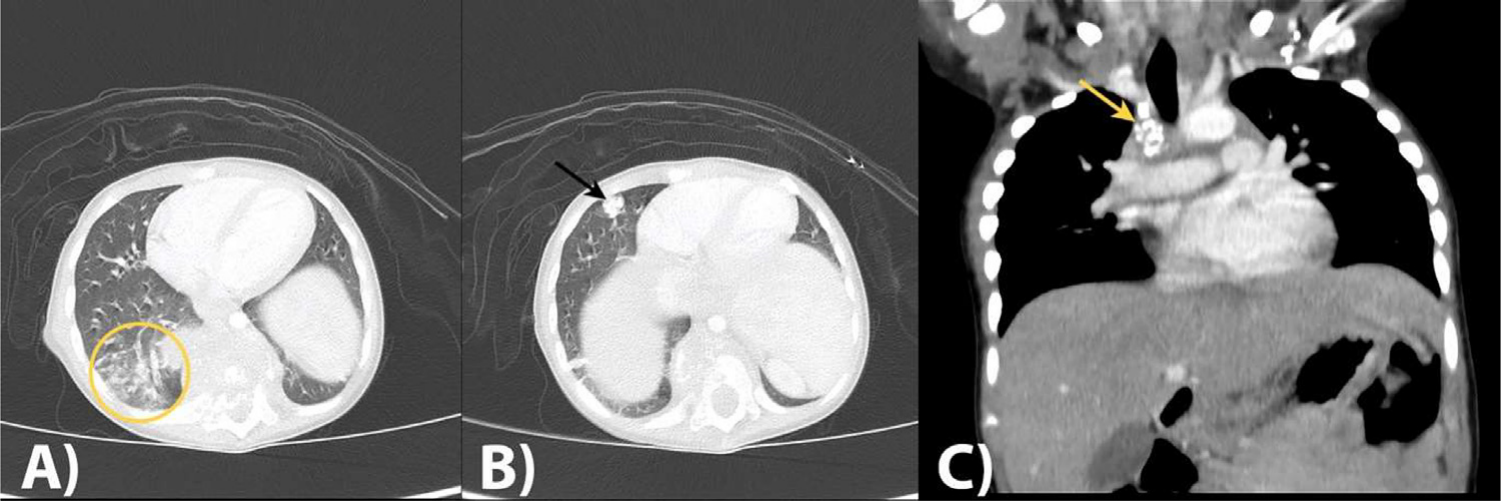
Contrast-enhanced CT scan of the thorax revealed a tree-in-bud opacity (yellow circle in A) with ground-glass opacity, along with multiple pulmonary fibrosis bilaterally in the lung window (A and B). Additionally, a calcified nodule (black arrow in B) was observed on segment 8 of the right lung, indicating the presence of tuberculoma, and mediastinal lymphadenopathy (yellow arrow in C), providing further confirmation for tuberculosis.

Subsequently, the patient was diagnosed with paraparesis caused by tuberculous spondylitis and paravertebral abscess. The clinicians modified the ATT regimen for the patient and opted for surgical intervention, necessitating additional imaging. A contrast-enhanced spinal MRI was conducted to further examine the spine. The result showed spondylodiscitis affecting the T8-10 vertebrae, which resulted in the destruction of the vertebral bodies and a kyphotic deformity at that level (Fig. 2). It also revealed the presence of a bilateral paravertebral abscess, which formed a fistula extending to the right erector spinae muscle and the subcutaneous soft tissue starting from the T3-11 level (Figs. 2A and C). Additionally, the anterior portion of the spinal canal at the T7-11 level was affected, and there was swelling of the bone marrow along the vertebral bodies of T8 and T10 (shown in Figs. 2B and C). The presence of pulmonary consolidations shown in the MRI also indicated the possibility of tuberculosis. Further examination with contrast-enhanced CT scan was also performed to further evaluate the lungs. The lung window revealed a tree-in-bud opacity with ground-glass opacity, along with multiple bilateral pulmonary fibrosis (Figs. 3A and B). Additionally, a calcified nodule was observed on segment 8 of the right lung, indicating the presence of tuberculoma, and mediastinal lymphadenopathy, providing further confirmation for tuberculosis (Figs. 3B and C).

**Fig. 4:**
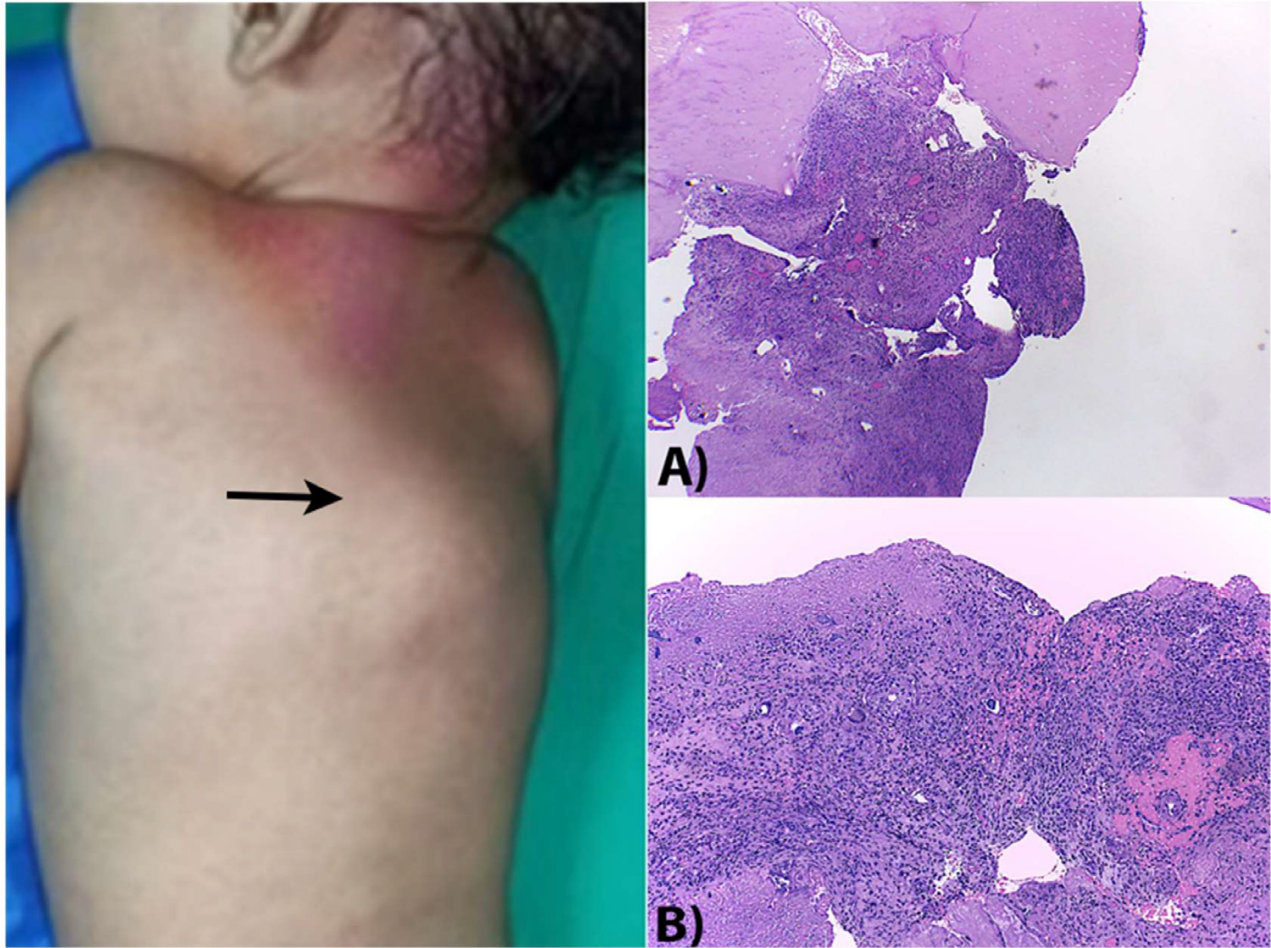
A kyphotic deformity with Gibbus was observed in the posterior thoracic region, accompanied by a mass on the midback region. The mass had a diameter of approximately 10 cm and was warm and painful to touch (black arrow). Surgical treatment was performed involving debridement and evacuation of the paravertebral abscess, as well as obtaining tissue samples for biopsy and culture. The biopsy findings in (A) (hematoxylin-eosin staining, 40 times magnification) and (B) (hematoxylin-eosin staining, 100 times magnification) showed the presence of chronic granulomatous inflammation, consistent with an infection caused by M. tuberculosis.

 ​The patient underwent surgical treatment involving decompression of the seventh, eighth, and ninth thoracic vertebrae by a thoracotomy approach. The procedure included debridement and evacuation of the paravertebral abscess, as well as obtaining tissue samples for biopsy and culture. The biopsy findings indicated the presence of chronic granulomatous inflammation, while the culture confirmed the presence of a MDR TB infection (Fig. 4). The ATT regimen was modified based on the patient's MDR status. The patient received treatment at our facility and had a steady improvement in her condition, leading to her discharge. Currently, the patient is in the seventh month of a long-term MDR TB regimen and is progressing satisfactorily.

## Discussion

Although there have been significant improvements in the diagnosis and treatment of tuberculosis (TB) in recent years, it continues to be a major global health issue, particularly in developing countries [3,5,9]. Children are one of the most susceptible group impacted by this disease, which leads to significant morbidity and mortality in pediatric populations, especially in countries with lower and middle incomes [3,5]. The primary factors that significantly contribute to the mortality rate among children include poverty, malnutrition, early age, female gender, widespread disease, co-infection with human immunodeficiency virus (HIV), and drug resistance [5]. Our case had a history of contact with her parents, who passed away from MDR TB infection. Additionally, malnutrition, a recognized risk factor, contributed to her immunocompromised state, making her more susceptible to infection.

The spine is frequently affected by TB in the skeletal system, which typically occurs as a secondary infection, when the bacteria spread hematogenously from the primary site of infection or through the Batson's venous plexus [8], [9], [10]. In children who are still growing, the disease can have extremely detrimental consequences, and these effects can continue to worsen even after the infection is brought under control [11]. The clinical onset is gradual and slow, with a prolonged duration and a resulting delayed presentation, which often consist of chronic back pain, tenderness, limited mobility, and non-specific symptoms [5,9,10]. These characteristics of tuberculous spondylitis were present in our patient, who sought treatment when the disease was well into an advanced stage. This is considering the initial clinical symptoms were nonspecific and develop gradually over time.

The thoracic spine is frequently affected in pediatric cases [2,3,9]. Mycobacterium is deposited in the anterior aspect of the vertebral endplates through the end arterioles [1,3,8]. Therefore, the anterior spinal involvement is the predominant manifestation of childhood tuberculous spondylitis, whereas posterior involvement is uncommon [8,9]. It commonly involves multiple vertebrae, typically affecting 2 or more contiguous vertebrae by the transmission of the disease through the vertebral artery [1,9]. Infection can also spread to the adjacent intervertebral disc, soft tissues, and subligamentous region. The intervertebral disc, being avascular, is not involved until the later stages of tuberculous spondylitis, as tuberculous bacilli, unlike pyogenic infection, do not produce proteolytic enzymes that affect the disc early on [3,[8], [9], [10]]. Soft tissue dissemination commonly manifests as a psoas abscess, which may subsequently manifest as discharging sinuses in unusual sites and when it expand into the epidural space, cause neurological deficits [5,8,10]. Kyphosis develops in patients with active tuberculous spondylitis as a result of the destruction of 3 or more vertebral bodies and subsequent collapse of the anterior column [8]. The collapse of multiple vertebrae, resulting in anterior wedging, causes the Gibbus deformity [5,9,10]. The presence of cartilage in the vertebral bodies of children makes them more susceptible to rapid deterioration of the vertebral bodies. This phenomenon can be attributed to the observation that abnormalities in children exhibit more severity and manifest at a faster rate in comparison to adults [8].

The diagnosis of tuberculous spondylitis is typically indicated by clinical features and imaging results [10]. Tissue diagnosis is considered the gold standard method for diagnosing tuberculous spondylitis, and confirmation of the presence of bacteria can be achieved using culture, histology, or polymerase chain reaction (PCR) [10]. Radiological imaging is a vital tool for diagnosing diverse TB manifestations, evaluating complications, monitoring treatment effectiveness, guiding interventions, and excluding alternative pathologies [5]. Plain radiographs are typically the initial diagnostic method used to investigate suspected cases of tuberculous spondylitis [2,5,9]. It offers a comprehensive view, assesses the level of involvement, and illustrates the pattern of an aggressive bone lesion [5]. Although conventional radiographs are widely used for diagnosis, especially in settings with limited resources, they have certain disadvantages [2,3,5,8]. At least 50% of the vertebral body must be damaged before changes are seen, which can take up to 6 months after infection. Early or subtle abnormalities may go unnoticed, thus further imaging is usually required if clinical suspicion persists [3,5,8].

Computed tomographic (CT) imaging provides improved visualization of irregular lytic lesions, sclerosis, disc collapse, disruption of bone circumference, paravertebral abscess, enhancing granulation tissue, and spinal canal disruptions by bone fragments, disc debris, or pus compared to plain radiography. However, CT cannot assess bone edema or provide detailed information about the spinal cord [3,9,10]. The CT scan effectively assesses the posterior elements, particularly the pedicles, which is crucial when considering surgery [5]. CT scans may be a beneficial alternative to MRI in resource-limited regions where TB is common, as they are more readily accessible. They can be particularly helpful in cases of acute myelopathy for surgical planning. However, it is important to consider the potential risks of radiation exposure, especially in children [3,8,9]. Magnetic resonance imaging (MRI) is the preferred imaging technique for diagnosing tuberculous spondylitis due to its ability to avoid ionizing radiation and provide detailed visualization of the spinal cord, intervertebral discs, and any soft tissue masses [2,8,9]. Additionally, it has the ability to detect tuberculous spondylitis 4-6 months earlier than conventional imaging techniques [5]. MRI often reveals disc collapse or destruction, cold abscess, vertebral wedging or collapse, marrow edema, and spinal abnormalities [3,8]. The vertebral bodies that are affected display hypointense signal (compared to muscle) on T1-weighted images and hyperintense signal (compared to muscle) on T2-weighted images, with inhomogeneous enhancement after the administration of contrast [3,8,9].

In complex cases like the one described in our findings, the use of multimodal imaging is crucial for understanding the anatomical abnormalities and providing an accurate diagnosis. Our case initially presented with ambulatory challenges and a mass on her midback, which were attributed to probable spinal TB. However, these symptoms progressed to paraparesis, prompting further evaluation and confirmation of tuberculous spondylitis with paravertebral abscess. The presence of bilateral paravertebral abscesses and a fistula extending to the right erector spinae muscle and the subcutaneous soft tissue, along with evidence of numerous vertebral body destruction and the presence of a Gibbus deformity, underscored the advanced stage of the disease in our patient. The extensive disease might also be attributed to the patient's immunocompromised state, which was a result of malnutrition and their low socio-economic status in a developing country.

The treatment necessitates a combination of ATT and careful utilization of surgery [8,11]. Surgery is typically considered for sudden or severe neurological deficits, progressive or unresponsive impairment despite non-surgical treatment, noticeable deformities, or involvement of both anterior, and posterior spine columns [8]. MDR-TB complicates the treatment of tuberculous spondylitis, affecting 11.7%-30.7% of culture-positive cases, because it may lead to increased surgical complications [11]. Due to the extent of the disease, presence of a neurological deficit, a large paravertebral abscess, and a deformity that could lead to future disability, the patient was not only treated with ATT, but also underwent surgical intervention. The surgery involved decompressing the T7-9 vertebrae through a thoracotomy approach, removing the abscess, and taking samples for biopsy and culture. The biopsy and culture results revealed the presence of an MDR TB, as a result, the ATT regimen was modified accordingly. Despite the MDR TB infection, our patient's case demonstrates the importance of comprehensive management strategies. The timely modification of the ATT regimen based on the patient's MDR status, together with surgical intervention, led to a successful outcome without complications. This highlights the critical role of early and comprehensive diagnosis, appropriate treatment, and multidisciplinary care in managing MDR-TB cases, particularly in pediatric patients. The child's condition improved sufficiently for her to be discharged post-surgery. She is currently in the seventh month of the MDR ATT regimen and showing clinical improvement.

## Conclusion

This case highlights the crucial role of radiological imaging in diagnosing and managing tuberculous spondylitis, especially in pediatric patients from developing nations facing challenges in TB control. The patient's age, complex medical history, and also parental death from MDR TB, underscores the socioeconomic factors contributing to the disease burden. The case also emphasizes the necessity of early radiological intervention, employing multimodality imaging for a comprehensive diagnostic approach to assess disease extent and morphology. Radiology played a pivotal role in promptly identifying tuberculous spondylitis, guiding timely management and surgical intervention. Maintaining a heightened awareness of TB's diverse imaging characteristics is essential for radiologists, emphasizing the collaborative efforts of radiologists and clinicians in developing nations to raise awareness, achieve early diagnosis, and ensure effective management.

## Patient consent

Written informed consent for the publication of this case report was obtained from the patient.

## CRediT authorship contribution statement

**Damayanti Sekarsari:** Conceptualization, Investigation, Resources, Writing – original draft, Writing – review & editing, Supervision. **Quinta Febryani Handoyono:** Writing – original draft, Writing – review & editing, Investigation. **Mohamad Yanuar Amal:** Writing – review & editing, Supervision. **Maria Angela Putri Maharani:** Resources. **Andra Hendriarto:** Resources.
